# An Image Registration Method for Colposcopic Images

**DOI:** 10.1155/2013/285962

**Published:** 2013-09-24

**Authors:** Efrén Mezura-Montes, Héctor-Gabriel Acosta-Mesa, Darío-del-Sinaí Ramírez-Garcés, Nicandro Cruz-Ramírez, Rodolfo Hernández-Jiménez

**Affiliations:** ^1^Department of Artificial Intelligence, University of Veracruz, Sebastián Camacho 5, 91000 Centro Xalapa, VER, Mexico; ^2^National Laboratory for Advanced Informatics (LANIA) A.C., Rébsamen 80, 9100 Centro Xalapa, VER, Mexico; ^3^Local Obstetrical & Gynecological College, Diego Leño 22, 91000 Xalapa, VER, Mexico

## Abstract

A nonrigid body image registration method for spatiotemporal alignment of image sequences obtained from colposcopy examinations to detect precancerous lesions of the cervix is proposed in this paper. The approach is based on time series calculation for those pixels in the first image of the sequence and a division of such image into small windows. A search process is then carried out to find the window with the highest affinity in each image of the sequence and replace it with the window in the reference image. The affinity value is based on polynomial approximation of the time series computed and the search is bounded by a search radius which defines the neighborhood of each window. The proposed approach is tested in ten 310-frame real cases in two experiments: the first one to determine the best values for the window size and the search radius and the second one to compare the best obtained results with respect to four registration methods found in the specialized literature. The obtained results show a robust and competitive performance of the proposed approach with a significant lower time with respect to the compared methods.

## 1. Introduction

Image registration is the process of overlaying two or more images of the same scene taken at different times, from different points of view or by different sensors. Such process then geometrically aligns two images (the reference and sensed images) and it is applied in different areas such as remote sensing, medicine, cartography, and computer vision [1].

The steps usually followed by a registration method consists of the following [2]: (1) manual or automated feature detection, (2) feature matching between the sensed image and the reference image, (3) mapping functions parameter estimation, and (4) image resampling and transformation (i.e., the sensed image is transformed by means of the mapping functions).

Image registration methods are quite diverse in the specialized literature because it is very difficult to design a universal method applicable to all registration tasks [3]. Every method should take into account not only the assumed type of geometric deformation among images but also radiometric deformations and noise corruption [4], required registration accuracy, and application-dependent data characteristics. Therefore, the selection of the most suitable method is dependent on the particular problem, the computational complexity of the evaluation criterion, and depends also on the desired precision of the results [1, 2, 4, 5]. Furthermore, approximated approaches such as evolutionary algorithms and metaheuristics have been adopted, mainly in the medical domain [6].

In this paper, the problem of interest is the spatiotemporal registration of image sequences which can be defined as follows: let *Ω* × *τ* ⊂ *ℜ* ^2^ × *ℜ* be the acquisition space-time of the reference time series of images *I*, and *Ω*′ × *τ*′ ⊂ *ℜ* ^2^ × *ℜ* the acquisition space-time in the target time series of images *I*′, with *Ω*: 2D space and *τ*: temporal domain.

When registering the target sequence *I*′ to the reference sequence *I*, the spatiotemporal transformation *S* that maps a spatiotemporal position (*x*, *t*) of *I* to the corresponding spatiotemporal position (*x*′, *t*′) of *I*′ must be found:

(1) $$\begin{array}{c} S:\Omega \times \tau \to \Omega^{\prime }\times \tau^{\prime },\\ \left(x,t\right)\to S\left(x,t\right)=\left(x^{\prime },t^{\prime }\right). \end{array}$$

The spatiotemporal transformation *S* is a combination of a spatial transformation *x*′ = *S*_space_(*x*, *t*) and the temporal transformation *t*′ = *S*_time_(*x*, *t*). We make the assumption that the temporal transformation *S*_time_ is time dependent: *t*′ = *S*_time_(*t*).

The particular case of image sequences considered in this work is related with the colposcopy examination to detect precancerous lesions of the cervix [7].

The colposcopic test consists of the evaluation of the level of white color intensity that the cervical tissue reaches after acetic acid application. This phenomenon is explained by the fact that the cells altered by cancer disease have bigger nucleus than the healthy cells. When acetic acid is absorbed by the damaged cell, it gets into the cell's nucleus, then the nuclear proteins contained in it are coagulated obtruding the pathway of light. This produces a reflection of light over the tissue that is perceived as a white area, whereas healthy tissue contains lower concentration of proteins in the nucleus, letting the light pass through the cytoplasm reaching the stroma [8].

The acquisition process of colposcopic images spans over 5 minutes and even though that the patient is fixed, and some small random movements are unavoidable. They have often local character (patients breathing, movements due to the muscle tonus, etc.). To be able to analyze the sequence of the images, the structures in the images should be brought into the same position by removing the differences due to the patient movements; that is, the colposcopic images have to be registered. This step is essential in this application; the goal of the 2D image spatial alignment is to enable comparison between corresponding anatomical positions.

The image sequences used in this work are taken with a colposcope and they are used as an input of a system designed to help in the prediagnose of cervical cancer. In fact, similar images have been used to test image segmentation approaches coupled with machine learning supervised learning methods [9].

The difficulty in the particular image registration problem aforementioned is that the cervix is undergoing a spatially and temporally varying degree motion during the video. Therefore, spatial alignment of corresponding frames of the image sequence is not sufficient since these frames may not correspond to the same temporal position in the video. This is mainly due to differences in the acquisition parameters and differences in the dynamic properties of the cervix. To tackle such problem we need to obtain the transformation model, a similarity criterion, and a search strategy.

This work precisely proposes a spatiotemporal alignment to enable comparison between corresponding anatomical positions with the aim to resolve spatial ambiguities which occur when there is not sufficient common appearance in the colposcopic images. The proposed approach is tested on a set of real subjects and it is also compared with respect to four registration methods found in the specialized literature.

The work on registration methods for colposcopic images is scarce. However, there are some methods such as that proposed by [10] where a search process based on a multifeature entropy similarity criterion is carried out. However, such search process requires derivative computing because indirect optimization methods were adopted.

The paper is organized as follows: Section 2 details the proposed registration method. After that, Section 3 presents the experimental design, the obtained results, and their corresponding discussions. Finally, Section 4 summarizes the conclusions obtained and points out the future work.

## 2. Temporal Method

**Figure 1:**
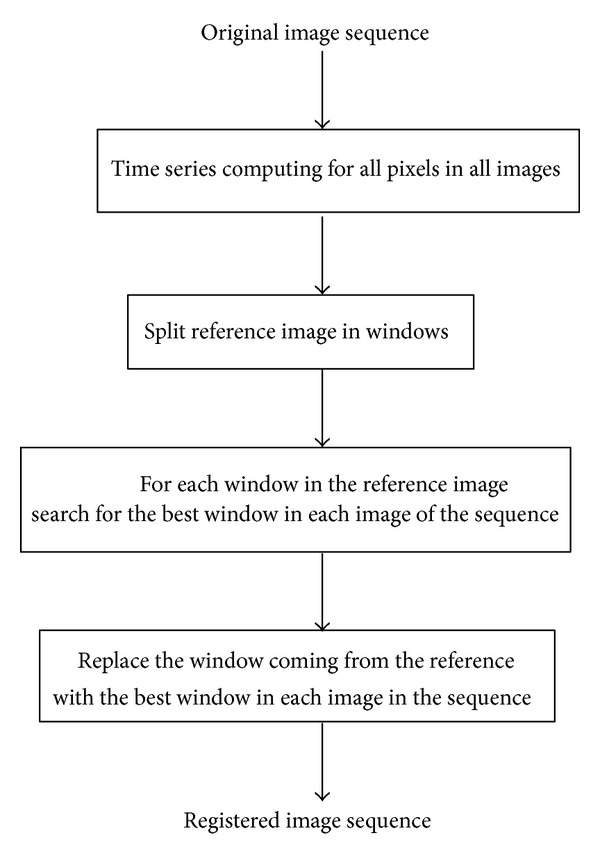
Proposed image registration method.

 The image registration method proposed, called Temporal method, is divided into different steps as it is presented in Figure 1. All images in the sequence are preprocessed by converting them from RGB to grayscale The first step calculates the times series for each pixel in the first image of the sequence. After that, the images are divided into windows and a search is carried out. Such search is based on the adjustment of each pixel's time series to a reference polynomial.

The process is detailed in the next subsections.

### 2.1. Time Series Calculation

The first step of the Temporal method consists in the calculation of the time series for each pixel in the first image in the sequence (also called reference image).

Considering the colposcopic sequence as an evolution of the intensity in time for each pixel, the temporal patterns can be extracted directly from the volume of images. Such patterns are called “Acetowhite response functions.” The image sequence can be represented as a volume of *t* 2D images *I*_*t*_(*x*, *y*), with the acquisition time *t* < *t* + 1. The color variation across the time in each pixel of the image gives a time series for each one of them. The sequence of resulting images can be seen as a block of 3D images *I*(*x*, *y*, *t*), defined in a spatiotemporal domain.

**Figure 2:**
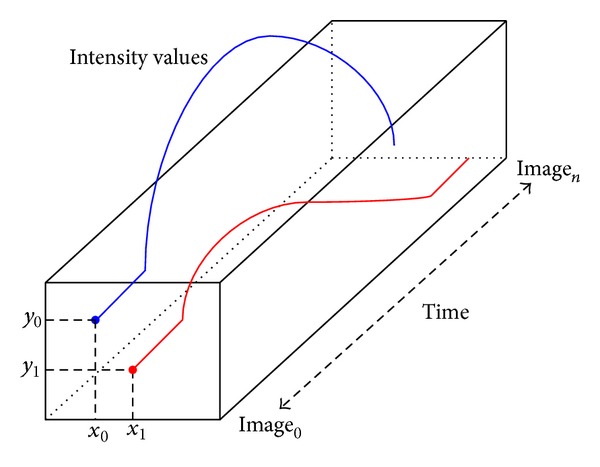
Time series computing of two pixels.

**Figure 3:**
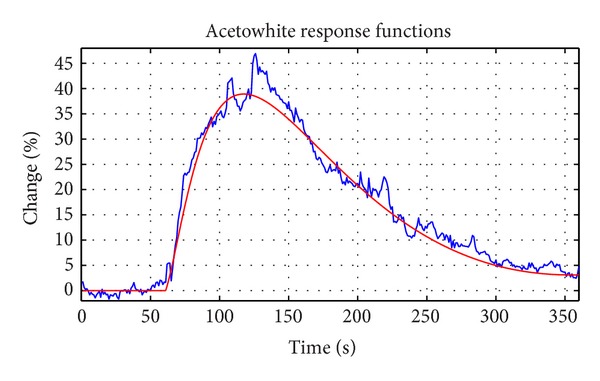
Time series of one pixel. The blue plot represents the values of the intensity of the pixel over time. The red line is the adjusted model explained later.

Figure 2 shows the acetowhite response functions, by following the change of the intensity of two pixels with coordinates (*x*_1_, *y*_1_) and (*x*_2_, *y*_2_), respectively, in the images sequence. The monitoring begins in the image 0 (i.e., reference image) and ends in image *n*, where *z* is the total number of images in the sequence (i.e., *z* represents the time). As a result, Figure 3 shows the time series plot of a pixel.

### 2.2. Window Generation

**Figure 4:**
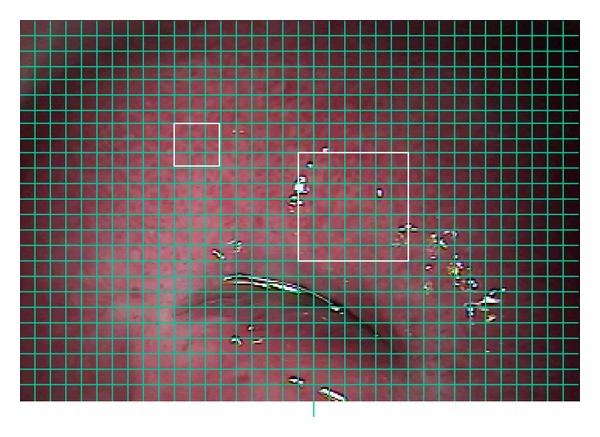
Reference image divided in windows.

Once the time series for all pixels have been computed, the next step consists of generating windows (i.e., small subsets of pixels) by using the reference image (see Figure 4).

### 2.3. Search Process

By using the windows generated in the reference image, a derivative-free search process starts with the aim to find the best correspondence of each window in the next image of the sequence. This search will be made according to the values of the time series of the pixels in each window and a user-defined parameter called *search radius*. The correspondence search for each window in the reference image must go through all the images in the sequence. This is the main contribution of this proposal, since the approaches found in the specialized literature consider only spatial information comparing two images, while the current approach takes advantage of the “*a-priori*” knowledge of the dynamics of the system, modeled using principal component Analysis (PCA) (see explanation below).

When such correspondence is found in all images, it is necessary to establish the same coordinates of the reference image for this window in all the sequence (i.e., generating the registered images).

The pseudocode of the search process is detailed in Algorithm 1. For each window in the reference image its center will be located in the next image in the sequence. After that, based on the search radius defined by the user, all neighbor-windows of the same size around the current window are generated. Then the quality values for the neighbor-windows, including the current window, are computed as follows.

1. Calculate the *s* time series in the window (*s* is the number of pixels in the window).
2. Compute the error value of each time series with respect to a polynomial approximation (explained in Algorithm 1).
3. Calculate the total error of the window by averaging the errors of all time series in the window.

The definition of the polynomial approximation used in the second step of the quality calculation for the neighbor-windows is as follows: each time series (acetowhite response function, AwRF) can be represented as a discrete-time multivariable stochastic system (see (2))

(2) $$\begin{array}{c} \text{AwRF}=\theta_{1}G_{1}+\theta_{2}G_{2}+\cdots +\theta_{n}G_{n}+e. \end{array}$$

**Figure 5:**
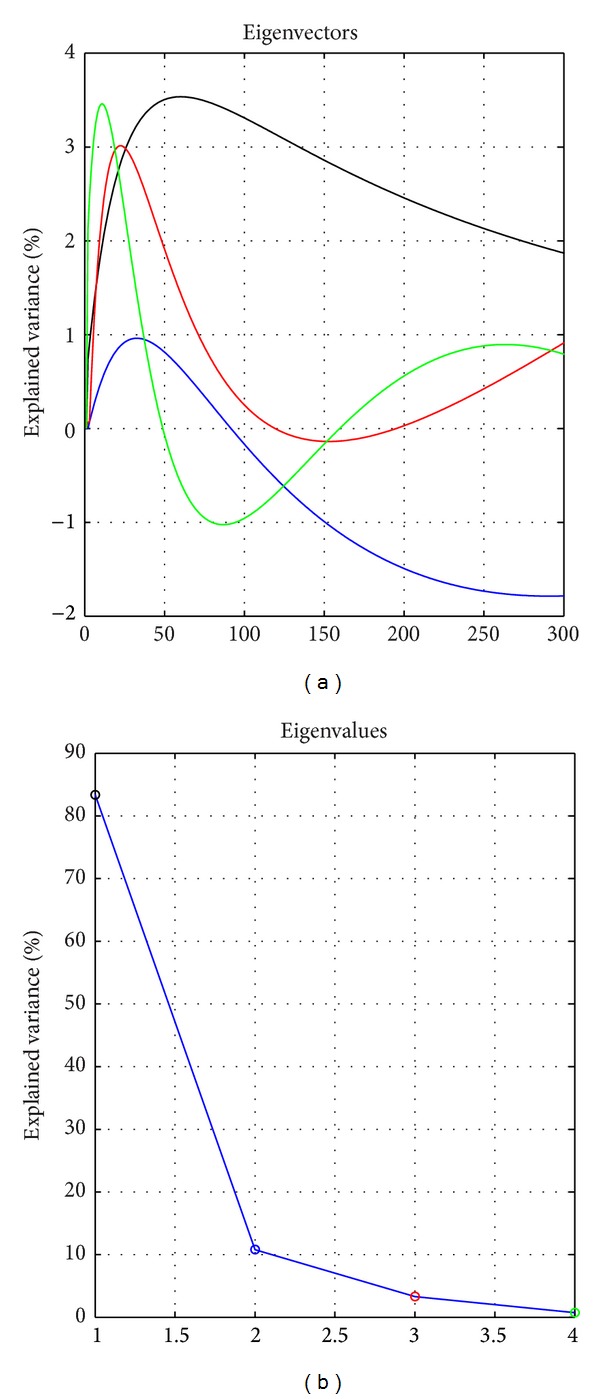
Four principal components and the corresponding eigenvalues.

In order to make a model to represent the general behaviour of the AwRF, a PCA analysis was developed over a set of representative AwRF. The objective of the analysis was to find the matrix *G* which explains the dynamics of every AwRF so as to filter high frequencies. The four principal components were selected to explain the 98.21% of the variance (see Figure 5).

Consider the linear model: AwRF*s* = *θ* *G* + *e*, where AwRFs is the temporal data matrix, *G* is the design matrix (in this case represented by the 4 first principal components computed as explained above and detailed in Figure 5), *θ* is the parameter matrix corresponding to the projections of AwRF in *G*, and *e* is the normally distributed noise vector.

**Figure 6:**
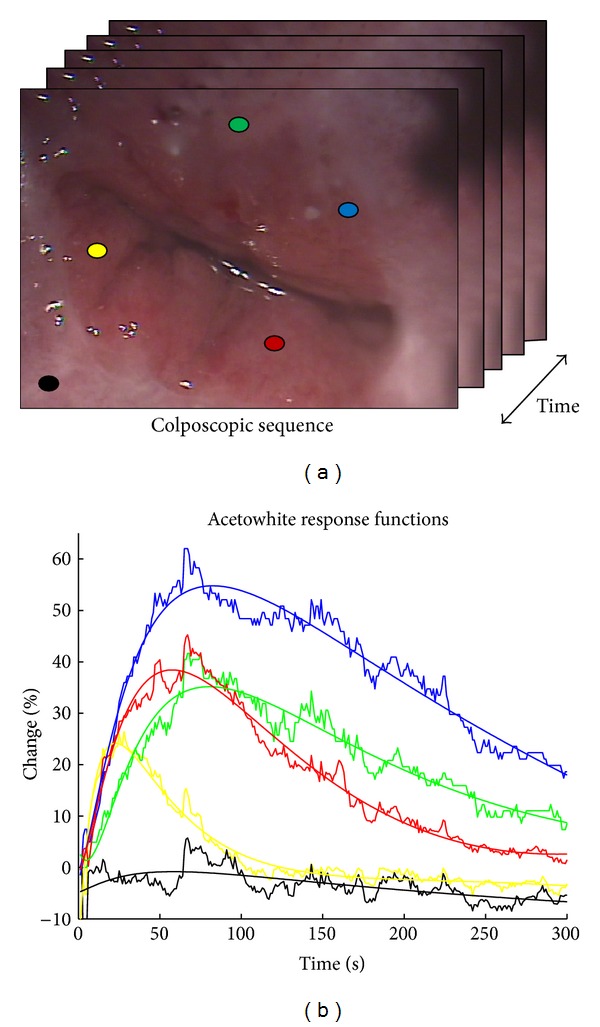
Time series extraction. The adjusted model is shown to its corresponding raw time series.

The parameter estimation can be done using a general linear approach in which the least square estimate of the parameter vector is indicated in (3) (see Figure 6):

(3) $$\begin{array}{c} \theta ={\left(G^{T}*G\right)}^{-1}*G^{T}*\text{AwRF}s. \end{array}$$

After all windows have been assigned their error values with respect to the polynomial approximation, the one with the lowest value is chosen and such window will be put in the location defined by the window coming from the reference image.

The aforementioned process is repeated for all windows in the reference image for all images in the sequence.

As it can be seen and recalling from Section 1, the proposed Temporal method has a transformation model (the window relocation based on error values), a similarity criterion (the average error per window in the neighborhood defined by the search radius and the polynomial approximation model), and a search strategy (Algorithm 1).

## 3. Experiments and Results

 The proposed Temporal method for image registration was tested in two experiments where representative real patient cases were considered. The first experiment consisted of an evaluation of the proposed approach by varying the window size and the search radius so as to obtain the most suitable values for such parameters. The second experiment was a comparison of the proposed approach against four image registration methods.

**Figure 7:**
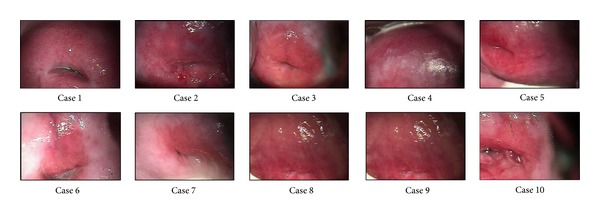
Ten real subjects (cases) used in the experiments.

Ten 310-frame real subject videos (cases) were used for comparison purposes (see Figure 7). Images were acquired using a colposcope dfv Vasconcellos model CP-M7 with magnification 16x without any optical filter. The viewing distance was 20 cm. and the field of view covers approximately a circle of 13 mm ratio. Images were acquired using a color camera Sony SSC-DC50A and a frame grabber Matrox Meteor-II/Standard driven by an HP workstation XW6000 running Matlab 7.0 image acquisition toolbox. During the first ten seconds of the image acquisition 10 images (352 × 240) were taken as baseline reference (1 frame/second); then after acetic acid application, three hundred images were taken in 5 minutes using the same sampling frequency. Control images taken at the beginning of each trial have a double purpose, the first one is to have a base reference to assess the signal percentage of change and the second one is to estimate the amount of signal noise. Each image was saved independently as a BMP file and was processed in gray scale. The computer used in the experiments was a HP Workstation XW6000 with 2 Intel Xeon processors at 2.66 GHz with 512 KB L2 cache and 2 GB of Registered ECC PC2100 DDR-266 memory. The version of Matlab was R2009a with Windows 7 as operative system. Due to the fact that the first 10 frames of the colposcopic sequence were captured just as a reference, they were discarded of the analysis. In all the experiments the mean square error (difference among the registered block of image sequence) is reported. 

### 3.1. Results of the First Experiment

**Figure 8:**
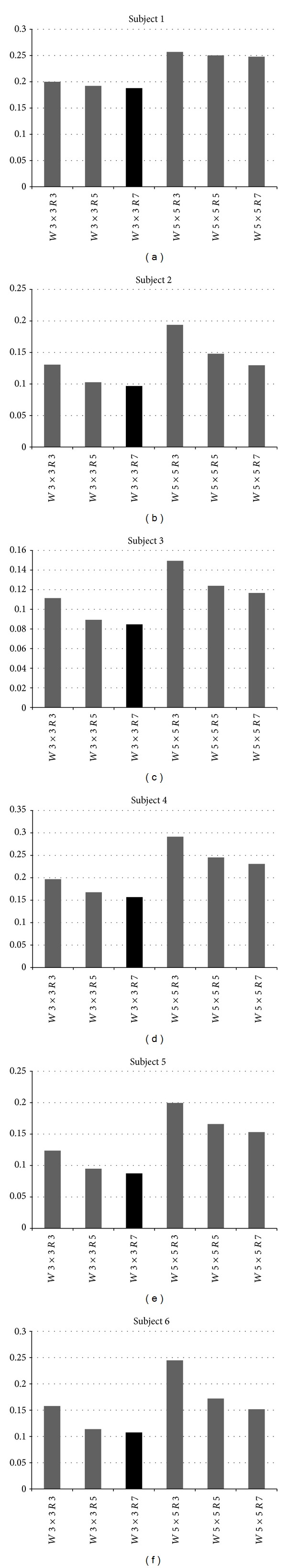
Results of experiment 1 (subjects 1 to 6). The error obtained by the Temporal method (*y*-axis) is shown for each combination of window size and search radius values (*x*-axis). Dark column is the one with the best (lowest) error.

**Figure 9:**
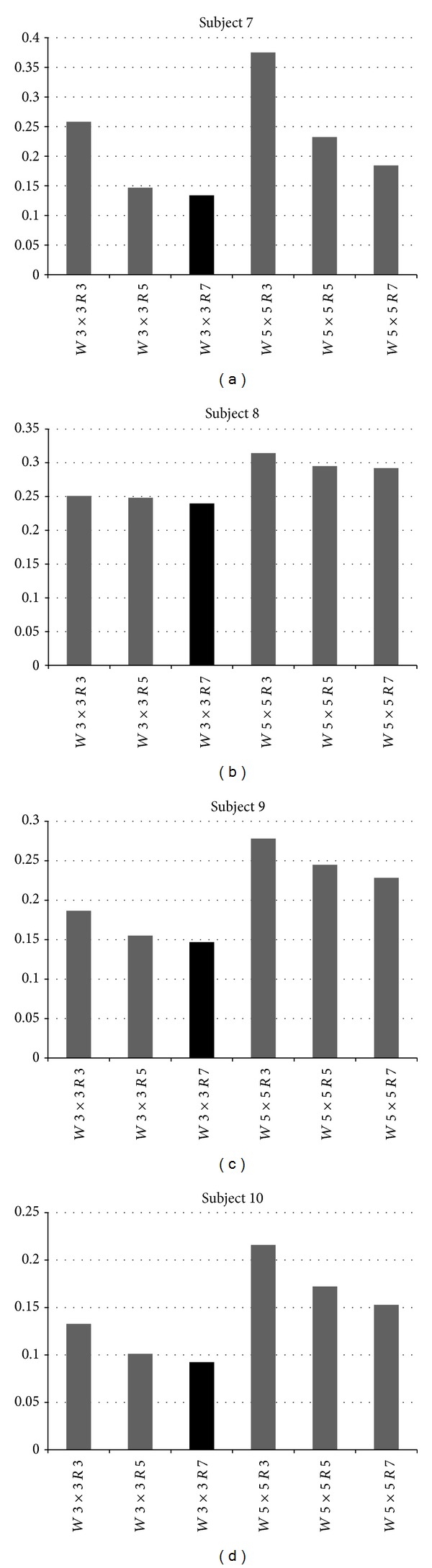
Results of experiment 1 (subjects 7 to 10). The error obtained by the Temporal method (*y*-axis) is shown for each combination of window size and search radius values (*x*-axis). Dark column is the one with the best (lowest) error.

Two window sizes (*W* = 3 × 3 and *W* = 5 × 5) and three search radius values (SR = 3, 5, 7) were tested in six combinations in the ten real subjects and the results are summarized in Figures 8 and 9. An interesting finding was observed in the results; in all ten subjects the combination *W* = 3 × 3 and SR = 7 provided the best results. Therefore, increasing the search radius combined with a small window size seems to provide competitive results. However, a larger search radius also increases the computational cost of the approach as the search becomes more intensive.

### 3.2. Results of the Second Experiment

In order to assess the performance of our proposal we compared the results obtained with the following well-known registration approaches:

B-Splines (the code was taken from http://www.mathworks.com/matlabcentral/fileexchange/20057-b-spline-grid-image-and-point-based-registration): this nonrigid registration algorithm corrects global motion using affine transformations while local motions is corrected by free-form deformations based on B-spline grids [11]. Registration is done using intensity-pixel-based features.

Fluid-Demon (the code was taken from http://www.mathworks.com/matlabcentral/fileexchange/21451-multimodality-non-rigid-demon-algorithm-image-registration): this method performs demon registration which is similar to non-rigid-fluid-like registration [12]. Motion is computed from intensity differences and gradient information and represented as a pixel velocity to correct the velocity field [13].

Subpixel (the code was taken from http://www.mathworks.com/matlabcentral/fileexchange/18401-efficient-subpixel-image-registration-by-cross-correlation): this algorithm corrects rotation and translation within a small fraction of a pixel using nonlinear optimization and matrix-multiply discrete Fourier transforms [14].

Mutual-Info (the code was taken from http://www.mathworks.com/matlabcentral/fileexchange/4145-automatic-image-registration-using-normalized-mutual-information-for-users-of-ip-toolbox): this algorithm corrects rotation and translation between images. Joint histogram computation is used as mutual information criteria [15]. The algorithm was run using a range of rotations from −10 to 10 with increments of 0.1 degree and translation of 10 pixels.

The best combination of parameters for the Temporal method, obtained in the first experiment (*W* = 3 × 3, SR = 7), was adopted to compare the results obtained with those reached by the four registration methods above mentioned (B-splines, Fluid-Demon, Subpixel, and Mutual-Info). The average error values per subject are presented in Table 1, while the corresponding standard deviation of the error values per subject is shown in Table 2. Moreover, the 95% confidence Kruskal-Wallis test was applied to verify the significance of the statistical values reported in Tables 1 and 2. Finally, the times required by each registration method on the ten subjects are summarized in Table 3. In all tables, the raw data was included as a reference value, in which case the mean square error was computed by subtracting consecutive frames from the sequence without any image processing.

Based on the Kruskal-Wallis test results, almost all differences observed in Tables 1 and 2 are significant with respect to the Temporal approach. 

**Figure 10:**
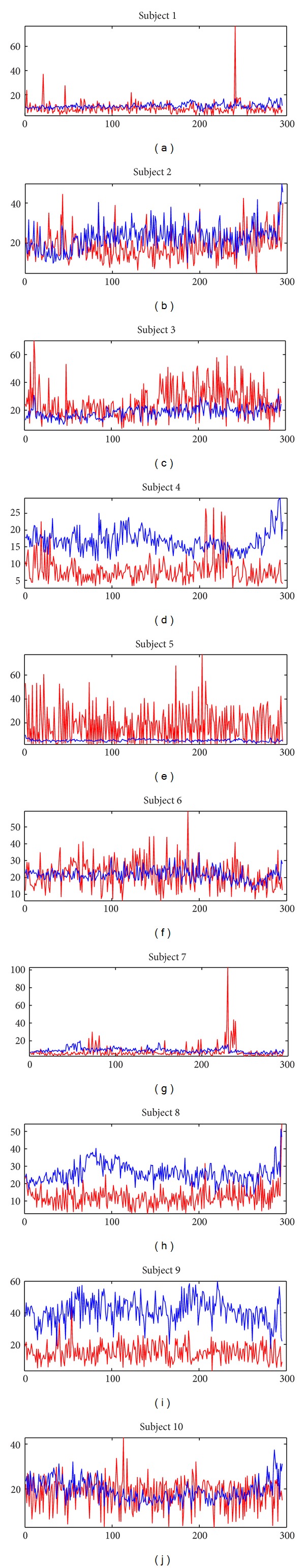
Error plotted frame by frame by the proposed Temporal method (blue line) and the B-splines method (red line).

The exceptions are the Fluid-Demon method in subject 8, and the B-splines method in subjects 6 and 10. Regarding the average error values per subject reported in Table 1, the most competitive methods were the B-splines (six lowest average error values) and the temporal approach (two lowest average error values). In the remaining two subjects both methods reached equally competitive values. In Figure 10 the frame-by-frame errors obtained in all ten subjects by B-splines and the Temporal approach are plotted.

Based only on the results reported in Table 1 and Figure 10, the Temporal method shows a competitive but not better performance with respect to the B-splines method. However, the standard deviation values in Table 2 suggest a more robust performance by the Temporal method over the B-splines method. Furthermore, in Table 3, the Temporal method reported a significant lower time with respect to the B-splines method. It is worth mentioning that the fastest method was Subpixel; however, their average and standard deviation error values in Tables 1 and 2 were outperformed by the proposed Temporal method in all but two subjects in the average error.

## 4. Conclusions and Future Work

A novel image registration method for spatiotemporal alignment of image sequences obtained from colposcopy examinations to detect precancerous lesions of the cervix, called Temporal method, was proposed in this paper. The proposed approach computed the time series for all pixels in the first image of the sequence because such values were the base to determine the similarity criterion later in the process. After that, the reference image was split into windows and for each window a derivative-free search process was carried out in all the sequence so as of finding the neighbor window with the highest similarity (the lowest average error based on a polynomial approximation). Such window in the reference image was then replaced in those high-affinity windows in each image of the sequence. The proposed approach was tested in ten 310-frame real subjects in two experiments: the first aimed to find the most suitable values for the window size and the search radius. It was found that a 3 × 3 window and a search radius of 7 consistently performed better in all test cases. Those results were compared in a second experiment with respect to four registration methods. The Temporal method provided the best tradeoff between low error values and time required to carry out the registration process. Furthermore, the Temporal method was the most robust among those methods compared. All results were statistically validated. The future work consists of analyzing a parallel version of the algorithm to further decrease the computational time required by this serial version and extending the registration of more real cases. Finally, adaptive mechanisms will be added to the approach for the window size and the search radius so as to keep the user from defining their values. 

## Figures and Tables

**Algorithm 1:**
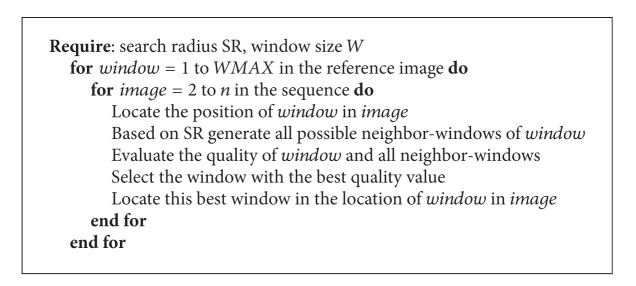
Search algorithm.

**Table 1:** Average error comparison per each subject against other registration methods.

Subject	Raw data	Fluid-Demon	Mutual-Info	B-splines	Subpixel	Temporal
1	44.87	18.32	54.84	**9.06 **	16.96	10.82
2	74.85	36.24	96.04	**18.37 **	42.83	22.68
3	67.91	49.82	91.48	25.42	54.07	**18.61 **
4	32.91	20.35	63.73	**7.96 **	21.50	16.78
5	35.69	29.54	70.81	18.19	17.67	**5.40 **
6	55.88	61.06	146.53	**21.71 **	38.03	**21.53 **
7	27.55	28.65	68.44	**7.37 **	24.78	9.17
8	49.16	27.39	86.62	**12.17 **	22.51	26.12
9	58.45	30.48	116.28	**15.01 **	18.24	41.23
10	61.56	44.89	110.01	**18.31 **	29.59	**19.19 **

In boldface those best results are remarked. If two results per case are considered as the best ones is because no significant difference between them was observed.

**Table 2:** Standard deviation of the error per each subject compared against other registration methods.

Subject	Raw data	Fluid-Demon	Mutual-Info	B-splines	Subpixel	Temporal
1	35.89	6.27	25.40	5.49	10.67	**2.00 **
2	44.17	10.46	33.89	6.83	22.02	**6.47 **
3	35.92	16.39	28.83	11.44	27.09	**3.98 **
4	34.63	9.55	22.46	3.75	25.53	**3.14 **
5	27.76	17.12	25.46	14.12	12.49	**0.92 **
6	32.18	27.88	28.22	7.91	18.68	**3.50 **
7	24.55	13.51	23.73	7.89	23.79	**2.67 **
8	40.98	11.95	32.90	5.56	16.62	**5.45 **
9	43.49	10.99	42.16	**5.76 **	8.23	7.87
10	47.59	19.24	29.49	6.50	15.96	**4.72 **

In boldface those best results are remarked.

**Table 3:** Time comparison (in seconds) of the proposed Temporal method against other registration methods.

Subject	Raw data	Fluid-Demon	Mutual-Info	B-splines	Subpixel	Temporal
1	0.08	29.10	23.74	105.45	**0.18 **	1.81
2	0.08	29.70	23.74	116.73	**0.18 **	1.54
3	0.08	32.96	23.46	97.75	**0.18 **	1.65
4	0.08	27.62	23.31	100.70	**0.18 **	1.35
5	0.08	30.34	23.39	103.33	**0.18 **	1.70
6	0.08	26.23	23.49	108.97	**0.18 **	1.70
7	0.08	33.66	23.23	107.32	**0.18 **	1.27
8	0.08	26.78	23.10	120.74	**0.18 **	1.60
9	0.08	24.96	23.06	125.98	**0.18 **	1.72
10	0.08	26.56	23.24	115.42	**0.18 **	1.62

Mean	0.08	28.79	23.38	110.24	**0.18 **	1.60
